# Supplementary material for: Bridges and Vertices in Heteroboranes
Source: Molecules. 2022 Dec 26;28(1):190. doi: 10.3390/molecules28010190 (PMC9822511; doi:10.3390/molecules28010190)
Supplement: Supplementary file 1 [file molecules-28-00190-s001.zip › molecules-2053280-supplementary.pdf]

Bridges and Vertices in Heteroboranes

Stuart A. Macgregor and Alan J. Welch

Supplementary Materials

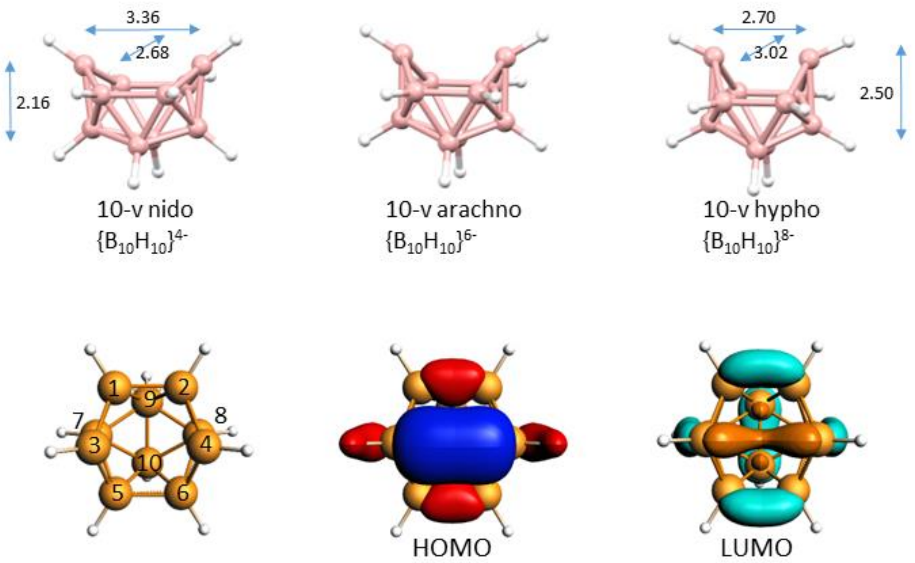

Figure S1. Upper row; key B to B dimensions in *nido* B{$_{10}$}, *arachno* {B_{10}} and *hypho* {B_{10}} fragments with the same topology. The fragments are constructed by removal of 1, 2 and 3 $\{BH\}^{2+}$ fragments from the DFT-optimized 11-, 12- and 13-vertex boranes $[B_{11}H_{11}]^{2-}$, $[B_{12}H_{12}]^{2-}$ and $[B_{13}H_{13}]^{2-}$ respectively. Lower row, left to right; atom numbering system, HOMO of $\{B_{10}H_{10}\}^{6-}$, LUMO of $\{B_{10}H_{10}\}^{6-}$. The changes in the dimensions of the three anions are easily rationalized by the nature of these molecular orbitals.

Table S1. Structure Overlay calculations (Å) between the experimental {B11} fragment of FAFYAN and the exemplar *nido* {B11} fragment (left) and the exemplar *arachno* {B11} fragment (right).

**FAFYAN / *n*-{B10}**		**FAFYAN / *a*-{B10}**	
1	0.047	1	0.046
2	0.157	2	0.078
3	0.037	3	0.018
4	0.023	4	0.028
5	0.161	5	0.082
6	0.089	6	0.028
7	0.198	7	0.092
8	0.490	8	0.089
9	0.195	9	0.097
10	0.315	10	0.128
11	0.299	11	0.114
**rms**	**0.227**	**rms**	**0.081**

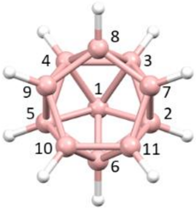

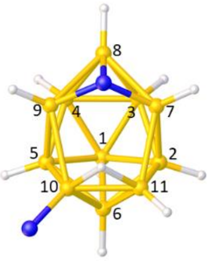

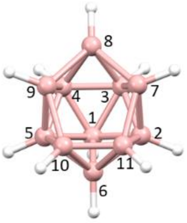

Table S2. Structure Overlay calculations (Å) between the experimental {B11} fragment of BUPPEI (primed cage) and exemplar *hypho* {B11} fragment (left) and exemplar *klado* {B11} fragment (right).

**BUPPEI / *h*-{B11}**		**BUPPEI / *k*-{B11}**	
1	0.065	1	0.156
2	0.067	2	0.154
3	0.114	3	0.245
4	0.101	4	0.258
5	0.029	5	0.094
6	0.044	6	0.058
7	0.058	7	0.046
8	0.034	8	0.032
9	0.025	9	0.037
10	0.054	10	0.215
11	0.021	11	0.018
**rms**	**0.063**	**rms**	**0.147**

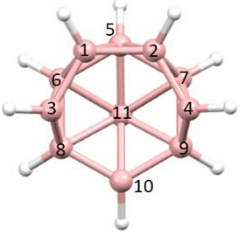

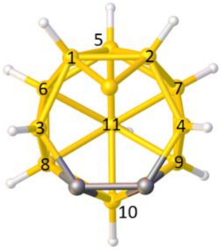

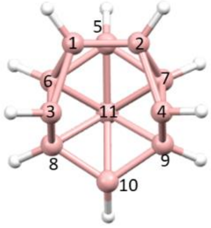

Table S3. Structure Overlay calculations (Å) between the DFT-optimized {B11} fragment of BUPPEI (center) and the experimental (crystallographic) {B11} fragments (left, unprimed cage; right, primed cage).

**DFT / unprimed**		**DFT / primed**	
1	0.011	1	0.024
2	0.015	2	0.024
3	0.024	3	0.027
4	0.020	4	0.019
5	0.023	5	0.023
6	0.021	6	0.009
7	0.022	7	0.019
8	0.025	8	0.017
9	0.019	9	0.017
10	0.013	10	0.005
11	0.009	11	0.008
**rms**	**0.019**	**rms**	**0.019**

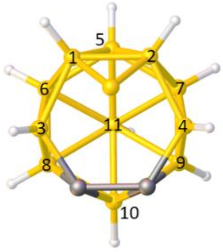

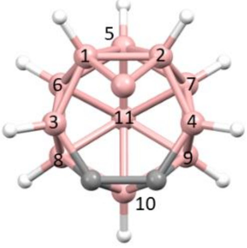

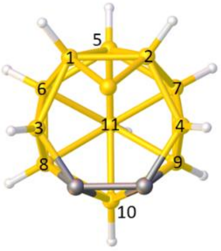

Computational Details.

All density functional theory (DFT) calculations employed the Gaussian16 [1] program. Geometries of *closo*-$[B_nH_n]^{2-}$ (n = 11-15) species were optimized with the BP86 functional [1,2,3] with the 6-31G** basis sets [4,5] applied to B and H atoms. This approach gave excellent agreement with the experimental structures of $[B_{11}H_{11}]^{2-}$ and $[B_{12}H_{12}]^{2-}$ (UQOBAE and LOSDIG respectively in the CSD) with rms misfits values of 0.021 Å and 0.009 Å respectively. Optimized structures were verified as true minima via analytical frequency calculations (all positive vibrational modes).

Table S4. Cartesian coordinates (Å) of all computed structures along with computed energies (hartrees) and lowest frequencies.

***closo*-$B_{11}H_{11}^{2-}$**

```
SCF  =          -280.075689321
H(0 K)=         -279.931184
H(298 K)=       -279.921458
G(298 K)=       -279.961914
Low Freq. = 172.9182cm-1, 308.0915cm-1

22
B  -0.91608  0.00000 -1.23925
B  -1.34076  0.93266  0.23092
B  -1.34076 -0.93266  0.23092
B   0.00000 -1.49159 -0.83735
B   0.91608 -0.00000 -1.23925
B   0.00000  1.49159 -0.83735
B   0.00000 -1.67796  0.92065
B   1.34076 -0.93266  0.23092
B   1.34076  0.93266  0.23092
B   0.00000  1.67796  0.92065
H  -1.63802  0.00000 -2.21840
H  -2.46619  1.37317  0.38651
H  -2.46619 -1.37317  0.38651
H   0.00000 -2.44978 -1.58886
H   1.63802 -0.00000 -2.21840
H   0.00000  2.44978 -1.58886
H   0.00000 -2.65943  1.63587
H   2.46619 -1.37317  0.38651
H   2.46619  1.37317  0.38651
H   0.00000  2.65943  1.63587
B   0.00000  0.00000  1.42002
H   0.00000  0.00000  2.63771
```

***closo*-$B_{12}H_{12}^{2-}$**

```
SCF  =          -305.650725475
H(0 K)=         -305.487957
H(298 K)=       -305.478847
G(298 K)=       -305.518896
Low Freq. = 515.7801cm-1, 515.7804cm-1

24
B  -0.00000  0.00000  1.70551
B   0.00000  1.52546  0.76273
B   1.45080  0.47139  0.76273
B   0.89664 -1.23412  0.76273
B  -0.89664 -1.23412  0.76273
B  -1.45080  0.47139  0.76273
B   1.45080 -0.47139 -0.76273
B  -0.00000 -1.52546 -0.76273
B  -1.45080 -0.47139 -0.76273
B  -0.89664  1.23412 -0.76273
H  -0.00000  0.00000  2.92072
H   0.00000  2.61237  1.30618
H   2.48451  0.80727  1.30618
H   1.53551 -2.11345  1.30618
H  -1.53551 -2.11345  1.30618
H  -2.48451  0.80727  1.30618
H   2.48451 -0.80727 -1.30618
H  -0.00000 -2.61237 -1.30618
H  -2.48451 -0.80727 -1.30618
H  -1.53551  2.11345 -1.30618
B   0.00000 -0.00000 -1.70551
B   0.89664  1.23412 -0.76273
H   0.00000 -0.00000 -2.92072
H   1.53551  2.11345 -1.30618
```

***closo*-$B_{13}H_{13}^{2-}$**

```
SCF  =          -331.037532076
H(0 K)=         -330.864255
H(298 K)=       -330.853079
G(298 K)=       -330.897095
Low Freq. = 141.3989cm-1, 234.1265cm-1

26
B  -1.28178 -1.40088  0.00034
B   0.26954 -1.51017  0.88510
B   0.99006  0.00122  1.43199
B   1.65577  0.89188 -0.00069
B   0.99001 -0.00095 -1.43201
B   1.65605 -0.89144  0.00064
B   0.26977 -1.51178 -0.88283
B  -0.84806 -0.00139 -1.35115
B   0.26909  1.51023 -0.88509
B   0.26935  1.51183  0.88285
B  -0.84787  0.00112  1.35126
B  -2.11969 -0.00027  0.00014
B  -1.28223  1.40060 -0.00054
H  -1.92256 -2.43951  0.00066
H  -1.36111 -0.00209 -2.45316
H  -1.92317  2.43913 -0.00095
H  -3.33426 -0.00057  0.00028
H   0.35560 -2.51295 -1.56422
H   0.35414  2.51021 -1.56832
H   0.35473 -2.51016  1.56831
H   0.35477  2.51304  1.56423
H   2.72016 -1.47537  0.00104
H   2.71972  1.47609 -0.00117
H   1.54654  0.00198  2.51094
H   1.54643 -0.00157 -2.51099
H  -1.36098  0.00176  2.45324
```

***closo*-$B_{14}H_{14}^{2-}$**

```
SCF  =          -356.545689060
H(0 K)=         -356.355939
H(298 K)=       -356.344839
G(298 K)=       -356.387884
Low Freq. = 328.1086cm-1, 329.2836cm-1

28
B   0.00000  0.00000 -1.57711
B   0.00000  1.75071 -0.76368
H   0.00000  2.80529 -1.36467
B   1.51622  0.87539 -0.76353
H   2.42944  1.40257 -1.36456
B   1.51622 -0.87539 -0.76353
H   2.42944 -1.40257 -1.36456
B   0.00000 -1.75071 -0.76368
H   0.00000 -2.80529 -1.36467
B  -1.51622 -0.87539 -0.76353
H  -2.42944 -1.40257 -1.36456
B  -1.51622  0.87539 -0.76353
H  -2.42944  1.40257 -1.36456
B   0.87538  1.51590  0.76362
H   1.40225  2.42925  1.36478
B   1.75120  0.00000  0.76348
H   2.80573  0.00000  1.36433
H   1.40225 -2.42925  1.36478
B   0.87538 -1.51590  0.76362
B  -0.87538 -1.51590  0.76362
H  -1.40225 -2.42925  1.36478
B  -1.75120 -0.00000  0.76348
H  -2.80573 -0.00000  1.36433
B  -0.87538  1.51590  0.76362
H  -1.40225  2.42925  1.36478
B   0.00000  0.00000  1.57709
H   0.00000  0.00000  2.78952
H   0.00000  0.00000 -2.78947
```

***closo*-$B_{15}H_{15}^{2-}$**

```
SCF  =          -381.995017068
H(0 K)=         -381.792125
H(298 K)=       -381.779913
G(298 K)=       -381.825587
Low Freq. = 301.7946cm-1, 302.2315cm-1

30
B   1.83083 -0.17932 -0.98215
B   1.83099 -0.76055  0.64628
H   2.75929 -1.35254  1.14969
B   1.83065  0.94044  0.33555
H   2.75888  1.67243  0.59678
B   0.84869 -1.59625 -0.57001
B   0.84846  0.30522  1.66716
H   1.27856  0.50893  2.78275
B   0.84827  1.29167 -1.09763
H   1.27781  2.15587 -1.83217
B   0.00068 -1.29631  1.10131
B  -0.00055  1.60146  0.57132
H  -0.00039  2.74293  0.97923
B  -0.00006 -0.30568 -1.67231
H   0.00013 -0.52315 -2.86466
B  -0.84853  0.30469  1.66776
H  -1.27866  0.50846  2.78327
B  -0.84891  1.29185 -1.09753
H  -1.27854  2.15607 -1.83194
B  -0.84857 -1.59657 -0.57031
H  -1.27834 -2.66469 -0.95148
B  -1.83049  0.93998  0.33571
H  -2.75925  1.67127  0.59714
B  -1.83091 -0.17969 -0.98192
H  -2.75959 -0.31933 -1.74592
B  -1.83037 -0.76104  0.64660
H  -2.75917 -1.35280  1.14943
H   0.00088 -2.21979  1.88624
H   1.27858 -2.66441 -0.95077
H   2.75896 -0.31869 -1.74675
```

BUPPEI

```
SCF  =          -634.349270328
H(0 K)=         -634.009185
H(298 K)=       -633.990367
G(298 K)=       -634.050749
Low Freq. = 68.4067cm-1, 95.2204cm-1

44
N  -2.94794  0.83558 -0.00029
C   1.28865  0.48829 -0.78103
C   1.28849  0.48842  0.78100
C   1.88393  1.83536 -1.22429
H   2.52062  1.72701 -2.11940
H   1.04535  2.50269 -1.49727
C   2.64658  2.39533 -0.00003
H   2.70077  3.49900 -0.00011
H   3.68240  2.01441  0.00009
C   1.88371  1.83554  1.22417
H   1.04510  2.50293  1.49692
H   2.52026  1.72731  2.11940
C  -3.34163  2.23407  0.00003
H  -2.44630  2.87373 -0.00029
H  -3.95558  2.48995  0.89299
H  -3.95636  2.49009 -0.89236
C  -4.09982 -0.05043 -0.00002
H  -3.77495 -1.09834 -0.00080
H  -4.73886  0.12210 -0.89386
H  -4.73779  0.12109  0.89478
B  -1.54582  0.45282 -0.00019
B   0.07732 -0.18156  1.67166
H  -0.11737  0.34828  2.73933
B  -1.21830 -1.15375  0.89901
H  -2.21165 -1.41877  1.53066
B  -1.21812 -1.15386 -0.89919
H  -2.21137 -1.41898 -1.53097
B   0.07768 -0.18184 -1.67180
H  -0.11683  0.34783 -2.73959
B   2.40691 -0.54972  0.00018
H   3.58670 -0.33155  0.00030
B   1.67671 -1.01019  1.50013
H   2.49181 -1.07750  2.38340
B   0.24713 -1.95142  1.50876
H   0.16609 -2.73675  2.41898
B  -0.47813 -2.48390  0.00007
H  -0.91985 -3.60351  0.00011
B   0.24736 -1.95163 -1.50861
H   0.16645 -2.73709 -2.41872
B   1.67701 -1.01048 -1.49981
H   2.49228 -1.07797 -2.38292
B   1.42031 -2.17552  0.00024
H   2.11364 -3.15899  0.00040
H  -0.85880  1.43982 -0.00031
```

Compound 1

```
SCF  =          -674.160581873
H(0 K)=         -673.778337
H(298 K)=       -673.758249
G(298 K)=       -673.821532
Low Freq. = 24.5546cm-1, 84.1154cm-1

48
N  -2.81187  0.63003  0.00397
C   1.45764  0.52288 -0.77128
C   1.45952  0.52191  0.77238
C   1.94350  1.90679 -1.22421
H   2.57859  1.84198 -2.12323
H   1.05689  2.50991 -1.49651
C   2.66656  2.51794  0.00016
H   2.64006  3.62035  0.00093
H   3.72630  2.21382 -0.00158
C   1.94698  1.90514  1.22578
H   2.58481  1.83881  2.12275
H   1.06132  2.50788  1.50170
C  -3.02288  1.38696 -1.27736
H  -2.27247  2.18647 -1.34995
H  -4.03840  1.81760 -1.29114
H  -2.89299  0.69285 -2.11964
C  -3.86862 -0.43574  0.09250
H  -4.85969  0.03925  0.00230
H  -3.78147 -0.95167  1.05597
H  -3.71620 -1.16046 -0.71677
B  -1.17688  0.17286  0.01011
B   0.20618 -0.22342  1.61406
H  -0.06560  0.30607  2.65962
B  -0.97643 -1.36364  0.90227
H  -1.94772 -1.65405  1.54863
B  -0.98002 -1.35976 -0.89259
H  -1.95679 -1.64374 -1.53755
B   0.20235 -0.22155 -1.60901
H  -0.07582  0.31125 -2.65111
B   2.61933 -0.44180 -0.00215
H   3.78141 -0.16639 -0.00348
B   1.88763 -0.94478  1.49474
H   2.66793 -0.94720  2.40479
B   0.54980 -1.99822  1.50627
H   0.54429 -2.77675  2.41771
B  -0.13093 -2.61937  0.00094
H  -0.48982 -3.76182  0.00054
B   0.54299 -1.99504 -1.50600
H   0.53215 -2.77193 -2.41884
B   1.88296 -0.94359 -1.49684
H   2.66114 -0.94561 -2.40873
B   1.72474 -2.13972 -0.00183
H   2.48759 -3.06366 -0.00374
H  -0.70496  1.27436  0.00063
C  -2.99713  1.56040  1.16924
H  -2.28765  2.39477  1.08123
H  -4.03168  1.94303  1.17820
H  -2.79010  1.00705  2.09651
```

References

1. Frisch, M.J.; Trucks, G.W.; Schlegel, H.B.; Scuseria, G.E.; Robb, M.A.; Cheeseman, J.R.; Scalmani, G.; Barone, V.; Petersson, G.A.; Nakatsuji, H.; Li, X.; Caricato, M.; Marenich, A.V.; Bloino, J.; Janesko, B.G.; Gomperts, R.; Mennucci, B.; Hratchian, H.P.; Ortiz, J.V.; Izmaylov, A.F.; Sonnenberg, J.L.; Williams-Young, D.; Ding, F.; Lipparini, F.; Egidi, F.; Goings, J.; Peng, B.; Petrone, A.; Henderson, T.; Ranasinghe, D.; Zakrzewski, V.G.; Gao, J.; Rega, N.; Zheng, G.; W. Liang; Hada, M.; Ehara, M.; Toyota, K.; Fukuda, R.; Hasegawa, J.; Ishida, M.; Nakajima, T.; Honda, Y.; Kitao, O.; Nakai, H.; Vreven, T.; Throssell, K.; Montgomery, J.A., Jr.; Peralta, J.E.; Ogliaro, F.; Bearpark, M.J.; Heyd, J.J.; Brothers, E.N.; Kudin, K.N.; Staroverov, V.N.; Keith, T.A.; Kobayashi, R.; Normand, J.; Raghavachari, K.; Rendell, A.P.; Burant, J.C.; Iyengar, S.S.; Tomasi, J.; Cossi, M.; Millam, J.M.; Klene, M.; Adamo, C.; Cammi, R.; Ochterski, J.W.; Martin, R.L.; Morokuma, K.; Farkas, O.; Foresman, J.B.; Fox, D.J. Gaussian 16, Revision A.03, Gaussian, Inc: Wallingford CT, 2016.
2. Becke, A.D. Density-Functional Exchange-Energy Approximation with Correct Asymptotic Behavior. *Phys. Rev. A* **1988**, *38*, 3098-3100.
3. Perdew, J.P. Density-Functional Approximation for the Correlation Energy of the Inhomogeneous Electron Gas. *Phys. Rev. B* **1986**, *33*, 8822-8824.
4. Hariharan, P.C.; Pople, J.A. The influence of polarization functions on molecular orbital hydrogenation energies. *Theor. Chim. Acta* **1973**, *28*, 213-222.
5. Hehre, W.J.; Ditchfield, R.; Pople, J.A. Self-Consistent Molecular Orbital Methods. XII. Further Extensions of Gaussian-Type Basis Sets for Use in Molecular Orbital Studies of Organic Molecules. *J. Chem. Phys.* **1972**, *56*, 2257-2261.
